# Extended Exhaled Nitric Oxide Analysis in Interstitial Lung Diseases: A Systematic Review

**DOI:** 10.3390/ijms21176187

**Published:** 2020-08-27

**Authors:** Paolo Cameli, Elena Bargagli, Laura Bergantini, Miriana d’Alessandro, Maria Pieroni, Giovanni A. Fontana, Piersante Sestini, Rosa Metella Refini

**Affiliations:** 1 Respiratory Diseases Unit, Department of Medicine, Surgery and Neurosciences, University of Siena, 53100 Siena, Italy; bargagli2@gmail.com (E.B.); laurabergantini@gmail.com (L.B.); dalessandro.miriana@gmail.com (M.d.); pieronim@unisi.it (M.P.); sestini@unisi.it (P.S.); refini@unisi.it (R.M.R.); 2 Department of Experimental and Clinical Medicine, University of Florence, 50121 Florence, Italy; giovanni.fontana@unifi.it

**Keywords:** idiopathic pulmonary fibrosis, interstitial lung disease, biomarker, exhaled breath, nitric oxide, oxidative stress, review

## Abstract

Fractional exhaled nitric oxide (FeNO) is a well-known and widely accepted biomarker of airways inflammation that can be useful in the therapeutic management, and adherence to inhalation therapy control, in asthmatic patients. However, the multiple-flows assessment of FeNO can provide a reliable measurement of bronchial and alveolar production of NO, supporting its potential value as biomarker also in peripheral lung diseases, such as interstitial lung diseases (ILD). In this review, we first discuss the role of NO in the pathobiology of lung fibrosis and the technique currently approved for the measurement of maximum bronchial flux of NO (J’awNO) and alveolar concentration of NO (CaNO). We systematically report the published evidence regarding extended FeNO analysis in the management of patients with different ILDs, focusing on its potential role in differential diagnosis, prognostic evaluation and severity assessment of disease. The few available data concerning extended FeNO analysis, and the most common comorbidities of ILD, are explored too. In conclusion, multiple-flows FeNO analysis, and CaNO in particular, appears to be a promising tool to be implemented in the diagnostic and prognostic pathways of patients affected with ILDs.

## 1. Background

The term fractional exhaled nitric oxide (FeNO) indicates a quantitative analysis of a gas, nitric oxide, in exhaled air. The presence of nitric oxide in exhaled air was first demonstrated through chemiluminescence analysis and mass spectrometry by Gustafsson in 1991 [1]. Nitric oxide plays a crucial role as an intra and extracellular mediator in many physiological and pathological processes such as modulation of vascular tone, thickening and remodeling of the bronchial muscle wall and in the regulation of local inflammation. The majority of cells resident in the respiratory system can produce NO, including pneumocytes I and II, bronchial epithelium, endothelium, smooth muscle cells and alveolar immune cells (macrophages, eosinophils and neutrophils). However, the production of NO is not univocal as different isoforms of NO-synthase can be expressed. In particular, inducible NO synthase (iNOS) is considered to contribute to more than 60% of NO production in the airways and is generally activated by immune and epithelial cells, triggered by inflammatory or infective processes [2,3].

Thus, the possibility to measure a biomarker of airway inflammation with a noninvasive, reproducible and economic technique led to the development of exhaled NO analyzers able to quantify the NO burden in the airways. In the last two decades, FeNO emerged as one of the most important biomarkers in the management of bronchial asthma, and the procedure for its measurement was first standardized in 2005 by ATS and ERS society [4]. Concerning clinical practice, FeNO is recommended by GINA documents as a tool for the prediction of exacerbations, the evaluation of response to treatment with inhaled corticosteroids (ICS) and the compliance to inhalation therapy. However, the reliability of FeNO in detecting NO-driven inflammation is limited to proximal airways, as the approved procedure for FeNO assessment consists of a 10 s steady expiration with a flow rate of 50 mL/s. Therefore, FeNO appears not to be reliable for the evaluation of NO production, and generally of inflammatory processes involving distal airways or alveolar spaces. Accordingly, conflicting results has been published concerning the significance of FeNO in respiratory diseases characterized by a more prominent involvement of peripheral districts, such as chronic obstructive pulmonary disease (COPD) or interstitial lung diseases (ILD) [5,6,7,8]. In order to overcome these limits, the extended analysis of FeNO, able to discriminate between bronchial and alveolar origins of eNO, have been repeatedly reported in the last decade, till the last technical standard document by ERS officially endorsed this procedure for future research [9].

Here, we perform a systematic review of literature in order to report all the available evidence concerning the rationale and the potential usefulness of extended FeNO analysis in the clinical management of ILDs.

**Figure 1:**
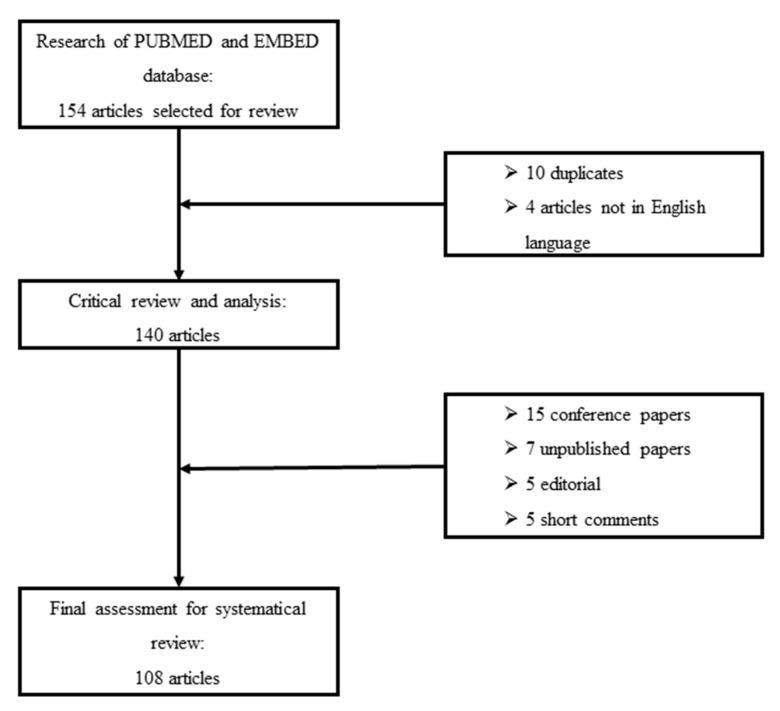
Flowchart of critical review and selection for inclusion of papers in the systematic review.

We perform a systematic search of PUBMED scientific papers in the English language. We used the keywords interstitial lung diseases, extended nitric oxide analysis, multiple-flows exhaled nitric oxide, and alveolar nitric oxide to discriminate potentially relevant studies. Reference lists of selected studies were also evaluated to obtain additional sources of evidence. We excluded conference papers, editorials, short letters and commentaries. Figure 1 shows the selection process for reviewed papers.

## 2. Nitric Oxide and Lung Fibrosis: Rationale

The role of NO in the pathogenesis and or pathophysiology of ILDs is not fully understood although oxidative stress is well recognized as an essential component for the development of lung fibrosis, particularly in idiopathic pulmonary fibrosis (IPF) [10,11]. The impacts of NO and related nitrosative stress on this field still need to be clarified. IPF is the most common and severe among idiopathic interstitial pneumonias (IIP), showing a typically progressive impairment of lung volume and diffusion capacity due to aberrant fibrogenesis and collagen deposition in the distal airspaces [12]. There are many enzymatic and immunological pathways that have been cited to contribute to the pathogenesis of IPF. However, to date, the primum movens of IPF is considered a genetically and epigenetically-influenced aberrant wound healing process in response to epithelial damage, leading to irreversible and progressive lung remodeling [13]. NO is considered a key mediator in the processes of epithelial wound healing and repairing, as the majority of cells involved in these mechanisms (platelets, fibroblast, epithelial and inflammatory cells) are able to produce NO through different pathways when activated [14,15,16,17]. Enzymatic inhibition, or gene deletion, of NOS isoforms significantly impair cell proliferation and angiogenesis, which are crucial in the epithelial reparation process [18,19]. The hypothesis that NO was also directly involved in lung fibrogenesis was first reported by Jung et al., who demonstrated an increased production of nitrate and nitrites in bronchoalveolar lavage (BAL) of bleomycin-induced lung fibrosis, associated with a significant overexpression of NOS2 [20]. However, these results were reported in murine models with acute lung injury after bleomycin challenge. Therefore, the increase of NO production could be still related to a nonspecific immunological response to a toxic agent. Pullamssetti et al. demonstrated on murine and human models of IPF an aberrant expression of dimethylarginine dimethylaminohydrolases (DDAHs) in fibrotic lungs, leading to uncontrolled activity of iNOS2 through the inhibition of asymmetric dimethylarginine (ADMA) [21]. The significance of this alteration was further confirmed by the inhibition of DDAH through a specific enzymatic inhibitor, which led to a reduction of epithelial proliferation and collagen production by resident fibroblasts in bleomycin-induced lung fibrosis [22]. Moreover, NO was reported to play a crucial role in the aberrant angiogenesis process of fibrotic lungs. In fact, Iyer and colleagues demonstrated that NO was the key molecule upregulating VEGF expression through the PI3k/Akt pathway [18]. These data were confirmed also by a proteomical approach [23] and are surely interesting as nintedanib, one of the approved pharmacological therapies for IPF, is a competitive nonspecific antagonist of VEGF, PDGF and FGF [24].

Substantial evidence suggesting the important role of NO in fibrogenesis has been published on systemic sclerosis-associated ILD (SSC-ILD). Despite its protective effect in terms of vasodilation, an overproduction of NO was demonstrated in affected tissues (skin and lung in particular), suggesting a dual activity of NO in different phases of disease [25]. This discrepancy may be determined by the different source of NO: eNOS seems to play a protective role against vascular remodeling, while iNOS was overexpressed by inflammatory cells, including alveolar macrophages, contributing to the development of interstitial fibrosis. Moreover, a study by Hua-Huy et al. reported that an increased production of NO by iNOS could even anticipate the appearance of lung fibrosis in SSC murine models, suggesting a potential role of NO not only in perpetuating, but also in triggering, the onset of interstitial lung involvement [26].

Still, the role of NO in lung fibrogenesis remains controversial. A recent paper by Noguchi et al. showed that triple knockout of the three isoforms of NOS (epithelial, neuronal and inducible) led to a significant deterioration of lung fibrosis that could be reverted with supplemental NO [27], suggesting a potential protective role of this molecule.

Therefore, more studies are needed to understand how the imbalance among different NOS can impact on the development of lung fibrosis, focusing on the potential protective/detrimental role of different patterns of enzymatic expression and/or specific cellular sources of NO.

## 3. Multiple Flows Feno Assessment: The Technical Approach

Many mathematical models have been proposed in the last two decades to properly discriminate and measure NO from different pulmonary districts [28,29,30]. All the suggested methodologies aimed to estimate bronchial and alveolar NO, propose new flow-independent parameters to discriminate NO production between proximal airways and more distal/alveolar spaces. The basic assumption was to incorporate the pulmonary architecture in a two-compartment model, considering the bronchial tree (from trachea to respiratory bronchioles) as a sort of rigid cylindrical tube and the acinar-alveolar compartment as an expansible part. This model is based on the hypothesis that, in basal conditions, there is a constant production of endogenous NO in both compartments. Consequently, the concentration of gas in the exhaled air (FeNO) depends on the capacity of NO to spread in alveolar or bronchial capillaries and on the expiratory flow [31]. Even if reproducible and somewhat reliable, the two-compartment model didn’t take into account the increasing surface area per unit volume of the bronchial tree (trumpet shape) and the axial diffusion of NO across the airways during the exhalation. For this reason, Condorelli et al. proposed a new model that considered these two variables, named as the trumpet model with axial diffusion (TMAD) that, through multiple exhalation flows for NO detection (at least three > 50 mL/sec), allowed the researchers to estimate the alveolar concentration of NO (CaNO) and the maximum airway flux of NO (J’awNO) [29]. This model has been endorsed in a technical standard paper by ERS on 2017 that recommended the online linear multiple-flows technique as the method of choice for extended exhaled NO analysis, and suggested the use of alveolar concentration of NO (CaNO) as a potential biomarker in distal airways or diffuse ILDs [9].

## 4. Extended Feno Analysis in Ilds: Current Evidence

ILDs are a very heterogeneous group including more than two hundred different histopathological disease entities. The majority of ILDs are rare, and the diagnosis is often a real challenge, requiring a multidisciplinary discussion and invasive diagnostic approaches in a relevant percentage of cases. Thus, scientific research has focused on the discovery and validation of biomarkers that could help clinicians in the diagnostic pathway to avoid more invasive and expensive exams. Among these biomarkers, extended FeNO analysis can be considered a very interesting tool because it is reproducible, easy to obtain, pain-free and relatively cheap. Therefore, many studies have been published to investigate its potential role in the clinical management of different ILDs. In Table 1, we list the studies focused on extended FeNO analysis in humans for any specific ILD included in this review.

### 4.1. SSC-ILD

SSC-ILD represents the most common among the subgroup of connective tissue disease-related ILDs (CTD-ILD). The early recognition and treatment of SSC-ILD is crucial in the management of these patients, as the development of lung fibrosis is a crucial prognostic factor because it constitutes the most common cause of death of SSC patients [56,57]. Moreover, SSC-ILD may show a wide variety of radiological patterns and accordingly different clinical courses, ranging from a substantial indolent disease to a very aggressive and rapidly progressive lung fibrosis, invariably leading to chronic respiratory failure and death. The evidence that nintedanib, an antifibrotic drug already approved for IPF treatment, can slow down lung disease progression in patients with SSC-ILD further enhances the urgent need of biomarkers for the early recognition of the presence of lung fibrosis, predicting disease progression and evaluating the response to treatment [58].

FeNO has been studied in SSC-ILD patients since 1997, reporting conflicting results due to the lack of standardization of sampling methods and the inclusion in study populations of patients with and without different lung comorbidities (reticular abnormalities, organizing pneumonia and pulmonary arterial hypertension) [59,60,61,62]. The first attempt to investigate bronchial and alveolar sites of production of NO was published in 2002, showing a significantly higher CaNO in patients with SSC-ILD and SSC-PH with respect to SSC with no lung involvement. Interestingly, CaNO inversely correlated with DLCO, suggesting the potential role of severity biomarker of CaNO, and the authors speculated that this correlation could be related to the impairment of diffusion capacity of the alveolar-capillary membrane [32]. Instead, in the following years, many studies hypothesized that the increase of CaNO in SSC-ILD patients was mainly determined by an increased production of NO in alveolar spaces, secondary to a pathological overexpression of iNOS in resident inflammatory cells. CaNO was found significantly increased also in patients with early lung fibrosis and experimental models demonstrated an aberrant activity of iNOS in a precox phase of lung fibrotic involvement, suggesting that NO could play a crucial role in the pathogenesis of SSC-ILD [26,33,34]. Moreover, CaNO was reported to be directly associated with serum-induced pulmonary fibroblast proliferation and myofibroblast rate of conversion, further strengthening the role of NO in the induction and progression of lung inflammation and fibrogenesis [35]. From a clinical point of view, extended FeNO analysis provided good reliability both to detect the presence of ILD [36] and to evaluate the severity of lung fibrosis in SSC patients, as CaNO reported significant correlations with respiratory functional parameters (TLC and DLCO) as well as CT quantitative score [37]. More interestingly, CaNO also showed a moderate to high accuracy in predicting the disease progression of SSC-ILD patients, as a 10% decrease in TLC or FVC from basal values in a 3-year period of follow-up, regardless of the functional parameters and/or the presence of ILD at baseline [38]. The same accuracy was reported, including mortality data in the statistical analysis, supporting a potential value of CaNO in predicting mortality risk in these patients. These results are interesting, as they suggest a critical value of this noninvasive and reproducible biomarker in the management of SSC-ILD patients who may show an unpredictable clinical course. On these assumptions, CaNO could be potentially helpful for clinicians to decide the initiation of specific treatment of lung fibrosis, or even to early identify those patients who will need lung transplantation. In this field, a study reported that SSC-ILD patients with higher CaNO values were more likely to respond to cyclophosphamide therapy, suggesting also a potential theranostic value of this biomarker [39]. To date, no data are available for other pharmacological treatments.

The current limitations of extended FeNO analysis in SSC-ILD are that no specific studies have compared CaNO with other prognostic biomarkers in SSC-ILD. As well, further studies are needed to properly assess the value of this biomarker in estimating survival in these patients.

### 4.2. IPF

Data concerning the potential role of multiple-flow analysis of FeNO in IPF has been growing in the last years. Although IPF is the most common and lethal among diffuse ILDs, diagnosis still represents a relevant challenge for clinicians, as well as the prediction of clinical course, that remains unpredictable [12,63]. A great number of biomarkers have been reported to be potentially useful in the diagnostic pathway and prognostic evaluation, but none of them has been universally approved in clinical practice [64,65,66,67,68,69]. The majority of proposed biomarkers come from blood, BAL or even histological samplings and, therefore, reveal an intrinsic weakness: on the one hand, the likely influence by concomitant conditions (e.g., diabetes, arterial hypertension, other therapies); on the other hand, the invasiveness of the procedure that may not be recommended in more fragile patients.

Thus, the possibility to obtain a reliable and noninvasive biomarker from exhaled breath, with a theoretically lower risk of sample contamination with respect to serum/blood, is surely intriguing.

The first evidence of an increased production of NO and derivates in IPF lungs was published in 1997, when Saleh and coauthors reported a significant overexpression of iNOS in inflammatory cells and alveolar epithelium associated with relevant production of nitrotyrosine compared to healthy subjects [70]. A higher amount of NO-derivate was also confirmed in BAL samplings [71,72], but extended exhaled NO assessment was first described in IPF patients only in 2011 by Schildge, who observed significant variations of CaNO among many ILD subgroups [40]. Few other studies have investigated the dynamics of multiple-flows exhaled NO in IPF, reporting, in the majority of cases, a significantly higher CaNO with respect to healthy subjects [41,42,43]. Only one study showed similar CaNO levels between IPF and healthy controls, but the study was limited by sample size, different smoking status and sex prevalence [54].

Concerning the association with pulmonary functional parameters, an inverse correlation between CaNO values and FVC and DLCO has been repeatedly observed, suggesting a potential value of this biomarker in the monitoring of disease progression [8,40,42,44,45]. One study also reported a significant correlation of CaNO with Composite Physiological Index (CPI), further supporting its potential as a severity biomarker in this setting [46]. Only two studies have specifically investigated the prognostic value of extended FeNO analysis in IPF, reporting a significantly worse survival and functional disease progression in patients with more elevated CaNO values [44,45]. No data are currently available regarding the dynamics of eNO parameters through the follow-up of IPF patients.

To date, no studies have been conducted to investigate the impact of antifibrotic treatment on CaNO values and if extended eNO analysis may be useful to predict or to quantify, the response to therapy in IPF patients.

### 4.3. Granulomatous Interstitial Lung Diseases

The only data available for extended FeNO analysis in granulomatous ILDs concerns two distinct disease entities: sarcoidosis and hypersensitivity pneumonitis (HP). Many studies have investigated the potential role of NO in the pathogenesis of sarcoidosis, reporting conflicting result. Although an overexpression of iNOS was demonstrated in sarcoid granulomas, significantly different than in other granulomatous disorders [47,73], measurements of BAL concentration of NO-derivates failed to show abnormal values. Conventional FeNO analysis in sarcoidosis showed controversial results, due also to different sampling methods of exhaled breath. The majority of papers didn’t report significant differences with respect to healthy controls [43,55,74,75], while the observations of higher levels of FeNO appeared not to be associated to disease activity, radiological stage, BAL cellular count or therapy status [76].

Multiple-flows eNO analysis was conducted in three studies [43,47,48], but CaNO didn’t report significant alterations. Among these studies, only Choi observed a weak but still significant inverse correlation between CaNO and DLCO [48], but these data were limited by the sample size and heterogeneity in terms of radiological stage and pulmonary involvement. A recent meta-analysis investigated the pooled evidence of eNO in patients with sarcoidosis, reporting substantially no significant differences compared to healthy controls and a high degree of heterogeneity of results, probably reflecting the complexity of sarcoidosis features and phenotypes [49]. No evidence is available concerning the potential role of eNO parameters as a severity or prognostic biomarker in sarcoidosis.

Regarding HP, very few and heterogeneous data are available, as eNO evaluation was conducted with different methods and in different phases of diseases (acute and chronic). Only two studies specifically assessed FeNO in patients with HP. Ojanguren et al. measured conventional FeNO before and after a specific inhalation challenge (SIC) to evaluate its potential role in diagnosis, reporting negative results [77]. Only one case report investigated CaNO in the same way, showing a significant increase of this biomarker after SIC [50]. Other studies included small cohorts of patients with HP in their eNO analysis, showing conflicting results: Guilleminault reported significantly higher levels of FeNO in HP patients than IPF, drug-related fibrosis and CTD-ILD [51], while two reports didn’t observe significant differences of FeNO and CaNO measurements with other ILDs [8,55].

### 4.4. Exposure-Related Lung Fibrosis

Extended eNO analysis has been specifically investigated in asbestos-related lung disease. A significant increase of CaNO values, but not of FeNO and bronchial NO levels, in patients with asbestosis was firstly reported in 2007, with the authors suggesting a correlation between asbestos-related alveolitis and CaNO [52]. Similar results were observed in a previous study by Sandrini et al., measuring FeNO with a exhalation flow rate of 200 mL/s [53]. These results were supported by the evidence in a murine model that alveolar macrophages were able to produce nitrite products through an overexpression of iNOS if exposed to asbestos [78]. These preliminary findings were subsequently confirmed by more recent studies [79,80] and further enhanced by the observation that iNOS genetic polymorphisms could influence the risk of developing lung fibrosis in asbestos exposed patients [81,82]. No specific data have been published on the accuracy of eNO parameters in detecting the onset of lung fibrosis in asbestos-exposed patients or their prognostic value.

### 4.5. Eosinophilic Pneumonia

Eosinophilic pneumonia is a rare inflammatory lung disorder characterized by a massive infiltration of eosinophils in alveolar spaces and interstitium; clinical aspects and radiological features are not very specific and usually BAL cellular count analysis is necessary to confirm diagnosis [83]. Considering that FeNO is considered a reliable biomarker of eosinophilic inflammation in asthmatic patients [84], a potential value of extended eNO assessment in EP can be speculated. Extended eNO analysis was investigated in patients affected with eosinophilic pneumonia (EP) in two studies [54,55]. Both papers reported similar values of FeNO and CaNO in these patients, including acute and chronic EP, that were significantly higher compared with other acute-onset ILDs (such as HP, sarcoidosis and cryptogenic organizing pneumonia) [55] or progressive fibrosing ILD (IPF) [54]. Interestingly, Furukawa et al. observed a strictly significant correlation between CaNO and the percentage of BAL cell actively expressing iNOS [54]. On a clinical point of view, Oishi et colleagues also reported relevant accuracy of combined FeNO and CaNO assessment to discriminate acute EP from other acute onset-ILDs, reporting a specificity of 100% for patients with FeNO > 23.4 ppb and CaNO > 10.2 ppb. Moreover, the degree of reduction of CaNO after corticosteroid treatment was significantly associated with reduction of peripheral blood eosinophil count, considered the most important biomarker of response to therapy in cEP, but not with improvement of lung functional parameters (FVC and DLCO) [55]. No data were reported concerning the prognostic significance of these findings.

### 4.6. The Value of Extended Eno Analysis in Differential Diagnosis of Ild

The potential value of eNO in the differential diagnosis of IPF is surely intriguing, but still largely unexplored. Few studies have been specifically designed to investigate the potential value of eNO parameters in the differential diagnosis of ILDs [7,8,51,55]. Among these, the papers by Oishi et al. and Cameli et al. were the only ones to implement multiple-flows eNO assessment in their studies. These two studies showed promising results of CaNO in the discrimination of a specific ILD or ILD subgroup from other diseases with similar clinical and radiological features. In particular, our research group observed a moderate to good accuracy of CaNO in discriminating CTD-ILDs (but not cHP) from idiopathic IIPs, while Oishi and colleagues reported a very high specificity of CaNO in identifying EP from other acute ILDs, such as cryptogenic organizing pneumonia, sarcoidosis and HP. No focused papers have been published to evaluate eNO parameters among different IIPs. However, our studies repeatedly failed to find any significant differences between IPF and idiopathic nonspecific interstitial pneumonia (NSIP) [8,42,43,45].

In summary, extended eNO assessment seems to have the potential to be implemented in the diagnostic pathway of ILDs, particularly to discriminate CTD-ILDs and EP, mainly due to its reproducibility, noninvasiveness and relatively low cost. However, multicenter and prospective studies are needed to confirm these findings and better evaluate its potential utility in this fie

## 5. Comorbidities/Complications of Ilds: A Pitfall for Eno Analysis?

### 5.1. Pulmonary Hypertension

Pulmonary arterial hypertension (PAH) is one of the most common complication of ILDs and is invariably associated with a significantly worse prognosis, quality of life and mortality [85,86,87]. Although it is more frequently diagnosed in patients with advanced fibrotic lung diseases, PAH may also be established in an earlier stage of disease, with patients showing exertional dyspnea out of proportion with respect to respiratory functional assessment or with signs or symptoms typical of cor pulmonale. The effect of ILD-related PAH on eNO parameters are largely unknown and mainly limited to SSc-ILD: published data of FeNO measurements in idiopathic PAH are conflicting [88,89,90]. Instead, CaNO appeared to be significantly increased in patients with PAH group 1 and group 3, accordingly, with impaired alveolar diffusion determined by endothelial remodeling [91,92,93]. Only one study reported CaNO values of a small cohort of patients SSc, including those with isolated ILD or PAH and their combination. No significant differences were found among these subgroups [93].

### 5.2. Obstructive Sleep Apnea

Many studies have investigated eNO behavior in patients with obstructive sleep apnea (OSA), reporting slightly, but still significantly higher levels of FeNO and J’awNO and, conversely, reduced CaNO values than healthy controls [94,95,96]. Interestingly, c-PAP therapy led to a normalization of eNO parameters, suggesting its potential role as a biomarker of compliance and response to therapy [97,98]. Although a significant percentage of ILD patients suffer from OSA [99,100], no data are currently available about eNO parameters in this setting. Despite lack of evidence, a significant influence of OSA on eNO parameters is likely and, therefore, quality of sleep should be assessed in ILD patients, considering also its negative prognostic impact [101].

### 5.3. Emphysema

The association of lower-lobe fibrosis with upper-lobe emphysema was first proposed as a distinct clinical entity and defined as combined pulmonary fibrosis and emphysema (CPFE) in 2005 [102]. To date, the debate on considering CPFE as a separate disease entity, or just a different phenotype of IPF, is still ongoing. However, considering the high percentage of PAH associated with CPFE, a potential influence on eNO parameters may be assumed [103]. To our knowledge, only one study performed a multiple-flows eNO comparison among patients with CPFE, emphysema and IPF, showing no differences between ILD subgroups. Moreover, patients with emphysema didn’t report significant modifications of CaNO with respect to healthy controls, suggesting that emphysema may not significantly influence CaNO values [41]. As a limitation, this study didn’t assess the presence of PAH and didn’t perform a CT quantitative analysis of emphysema extension to better match different subgroups of patients.

### 5.4. Lung Cancer

A higher prevalence of lung cancer in patients affected with ILD with respect to the general population has been repeatedly reported. However, it is still a matter of debate if ILD (particularly IPF) increases per se the probability of lung cancer development or is just related to the overlapping of risk factors associated with both diseases (e.g., smoking exposure, male sex, age > 60 years) [104,105,106,107]. Higher values of FeNO have been observed in patients with lung cancer, as well as an aberrant overexpression of iNOS by alveolar macrophages [108], but no data are currently available on extended eNO analysis.

## 6. Conclusions

In this review, we report the published evidence concerning the potential value of extended FeNO assessment in the management of patients with ILDs. Multiple-flows FeNO analysis is surely an interesting technique that could allow respiratory physicians to obtain a reproducible and inexpensive biomarker for the management of diffuse lung diseases. The standardization of technique, and the endorsement of ATS/ERS for extended FeNO as a source of biomarker, have boosted and ameliorated the quality of studies on this topic, although they are still few and monocentric. Among ILDs, many specific disease entities may show a progressive clinical course similar to IPF, leading to chronic respiratory failure and death in a few years. However, no biomarker has been widely accepted in the clinical practice in both diagnostic and prognostic estimation. In this field, CaNO has demonstrated an intriguing potential in discriminating idiopathic ILDs from CTD-ILDs and in estimating survival and disease progression in terms of FVC deterioration, suggesting its possible implementation in the clinical management of IPF due to its reproducibility, repeatability and noninvasive nature. However, these promising findings need to be furtherly confirmed by larger, prospective and multicenter studies to better investigate the dynamics of eNO parameters throughout the clinical course of disease and to evaluate the influence on CaNO played by comorbidities and antifibrotic treatment.

## Figures and Tables

**Table 1:** Characteristics of included studies focusing on the evaluation of extended FeNO analysis in humans for different ILDs. SSC-ILD: systemic sclerosis associated interstitial lung disease; PAH: pulmonary arterial hypertension; CaNO: alveolar concentration of nitric oxide; FeNO: fractional exhaled nitric oxide; DLCO: diffusion lung capacity for carbon monoxide; HC: healthy controls; SP-D: surfactant protein-D; TLC: total lung capacity; FVC: forced vital capacity; IPF: idiopathic pulmonary fibrosis; CTD-ILD: connective tissue disease associated interstitial lung disease; NSIP: non-specific interstitial pneumonia; cHP: chronic hypersensitivity pneumonitis; COP: cryptogenic organizing pneumonia; RB-ILD: respiratory bronchiolitis associated interstitial lung disease; IVC: inspiratory vital capacity; CPFE: combined pulmonary fibrosis and emphysema; 6MWD: 6-min walking distance; CPI: combined physiological index.

Author (Year)	Study Type	Sample Size	Principal Results	Ref
*SSC-ILD* Girgis et al. (2002)	Observational, cross-sectional study	20 (15 with SSC-ILD and 5 with SSC-PAH) 20 HC	➢ Higher CaNO values in SSC patients than HC ➢ Significant negative correlation between CaNO and DLCO	[32]
Wuttge et al. (2010)	Observational, cross-sectional study	34 (19 with SSC-ILD and 14 without ILD) 26 HC	➢ Higher CaNO values in SSC patients than HC ➢ Significant direct correlation between CaNO and radiological extension of ground glass opacities and reticulation	[33]
Benfante et al. (2018)	Observational, cross-sectional study	15 SSC 10 HC	➢ Higher CaNO values in SSC patients than HC ➢ Significant direct correlation between CaNO and serum SP-D concentrations	[34]
Hua-Huy et al. (2010)	Observational, cross-sectional study	37 (16 with SSC-ILD and 21 without ILD) 10 HC	➢ Higher CaNO values in SSC patients than HC ➢ CaNO > 4.3 ppb was associated with higher myofibroblast conversion induced by SSC serum ➢ Significant direct correlation between CaNO and pulmonary fibroblast proliferation induced by SSC serum	[35]
Tiev et al. (2009)	Observational, cross-sectional study	65 (38 with SSC-ILD and 27 without ILD)	➢ Accuracy of CaNO as biomarker of ILD detection: sensitivity 87% and specificity 59%	[36]
Tiev et al. (2007)	Observational, cross-sectional study	58 (33 with SSC-ILD and 23 without ILD) 19 HC	➢ Higher CaNO values in SSC-ILD patients than SSC without ILD and HC ➢ Significant inverse correlation between CaNO and lung functional parameters (TLC and DLCO) ➢ Significant direct correlation between CaNO and CT scan fibrosis score	[37]
Tiev et al. (2012)	Observational, 3-y longitudinal study	153 (74 with SSC-ILD and 79 without ILD)	➢ CaNO > 5.3 ppb was associated with a higher risk of decline of FVC or TLC and/or death (HR: 6.06, p<0.0001)	[38]
Tiev et al. (2014)	Open-label, monocentric uncontrolled trial	19 SSC-ILD	➢ CaNO > 8.5 ppb was associated with a good response to cyclophosphamide	[39]
*IPF* Cameli et al. (2019)	Observational, retrospective study	134 patients with ILD (50 IPF, 46 CTD-ILD, 19 NSIP and 19 cHP) 60 HC	➢ Higher CaNO values in all ILD patients than HC ➢ The highest CaNO values were observed in CTD-ILD patients ➢ Accuracy of CaNO for detection of CTD in ILD patients: sensitivity 60% and specificity 80% (cut-off: 13.09 ppb)	[8]
Schildge (2011)	Observational, cross sectional study	83 patients with ILD (14 IPF, 33 sarcoidosis, 12 CTD-ILD, 8 COP, 10 cHP and 6 RB-ILD) 17 HC	➢ Significant differences of CaNO between ILD subgroups and HC ➢ Significant negative correlation between CaNO and IVC	[40]
Zhao et al. (2012)	Observational, cross sectional study	14 patients with IPF, 22 with CPFE and 22 with emphysema 12 HC	➢ Higher values of CaNO in patients with IPF and CPFE than HC and emphysema subgroup	[41]
Cameli et al. (2014)	Observational, cross-sectional study	30 patients with ILD (22 IPF and 8 NSIP) 30 HC	➢ Higher values of CaNO and FeNO 50-100-150 and 350 mL/s in patients with IPF and NSIP than HC ➢ Significant correlations of CaNO with 6MWD, TLC, FVC and DLCO	[42]
Cameli et al. (2016)	Observational, retrospective study	53 patients with ILD (31 with sarcoidosis and 22 with IPF) 30 HC	➢ Higher values of CaNO and FeNO 50-100 and 150 mL/s in patients with IPF than HC and sarcoidosis	[43]
Kotecha et al. (2016)	Observational, prospective study	27 patients with IPF	➢ Higher values of CaNO were associated with a shorter time to disease progression and/or death	[44]
Cameli et al. (2019)	Observational, retrospective study	88 patients with IPF 60 HC	➢ Higher values of CaNO, FeNO 100-150 and 350 mL/s in IPF patients than HC ➢ Significant inverse correlation of CaNO with DLCO ➢ CaNO > 6 ppb was associated with a worse mortality ➢ CaNO > 9 ppb was associated with a shorter time to decline of FVC 10%	[45]
Cameli et al. (2019)	Observational, retrospective study	59 patients with IPF 60 HC	➢ Higher values of CaNO and FeNO 150-350 mL/s in IPF patients than HC ➢ Significant correlations between CaNO and DLCO and CPI ➢ Significant correlation between CaNO and serum periostin concentrations ➢ Patients with CaNO > 6 ppb showed a trend towards a worse mortality	[46]
*Granulomatous ILDs* Moodley et al. (1999)	Open-label, monocentric study	12 patients with sarcoidosis 21 HC	➢ Higher values of FeNO 250 mL/s in sarcoidosis than HC ➢ FeNO 250 mL/s values significantly reduced after steroid therapy	[47]
Choi et al. (2009)	Observational, cross-sectional study	42 patients with sarcoidosis 20 HC	➢ No differences of CaNO between sarcoidosis and HC groups, as well as between patients with active or inactive disease ➢ Significant inverse correlations of CaNO with FVC and DLCO	[48]
Terrington et al. (2019)	Systematic review and meta-analysis	4 studies included for analysis	➢ No differences of exhaled NO parameters between sarcoidosis and HC	[49]
Shirai et al. (2010)	Case report	1 patient with HP	➢ Significant increase of CaNO values after specific environmental challenge	[50]
Guilleminault et al. (2013)	Observational, cross-sectional study	61 patients with ILD (18 IPF, 13 HP, 22 CTD-ILD, 8 drug-induced ILD)	➢ Higher FeNO 50 values in HP patients that IPF, CTD-ILD or drug-induced ILD patients	[51]
Exposure-related *ILDs* Lehtonen et al. (2007)	Observational, cross sectional study	15 patients with asbestosis 15 HC	➢ Higher CaNO values in asbestosis patients than in HC	[52]
Sandrini et al. (2006)	Observational, cross-sectional study	56 patients with asbestos-related diseases (12 asbestosis, 32 pleural plaques, 12 diffuse pleural thickening) 35 HC	➢ Higher FeNO 200 mL/s values in patients with asbestosis and pleural plaques than HC and patients with diffuse pleural thickening)	[53]
*Eosinophilic pneumonia* Furukawa et al. (2011)	Open-label, monocentric uncontrolled study	56 patients with asbestos-related diseases (12 asbestosis, 32 pleural plaques, 12 diffuse pleural thickening) 35 HC	➢ Higher CaNO values in patients with eosinophilic pneumonia than IPF and HC ➢ CaNO reduction after steroid therapy was associated with decrease of blood eosinophils cell count	[54]
Oishi et al. (2017)	Observational, cross sectional study	40 patients with ILD (18 with acute eosinophilic pneumonia, 14 with COP, 5 with sarcoidosis and 3 with HP)	➢ Higher CaNO and FeNO 50 values in patients with acute eosinophilic pneumonitis than other acute-onset ILDs	[55]
